# Supplementary material for: Identifying the wide diversity of extraterrestrial purine and pyrimidine nucleobases in carbonaceous meteorites
Source: Nat Commun. 2022 Apr 26;13:2008. doi: 10.1038/s41467-022-29612-x (PMC9042847; doi:10.1038/s41467-022-29612-x)
Supplement: Supplementary file 1 — Supplementary Information [file 41467_2022_29612_MOESM1_ESM.pdf]

Supplementary Information for

Identifying the wide diversity of extraterrestrial purine and pyrimidine nucleobases in carbonaceous meteorites

Yasuhiro Oba, Yoshinori Takano, Yoshihiro Furukawa, Toshiki Koga,

Daniel P. Glavin, Jason P. Dworkin and Hiroshi Naraoka

Contents

- Supplementary Notes
- Supplementary Figures 1–13
- Supplementary Tables 1–3
- Supplementary References 1–30

Supplementary Note

Amino acids and amines with their analogues in the meteorite extracts

To compare with the series of N-heterocyclic compounds (-C-N-C-), we also searched linear N-containing compounds (-C-NH_2), namely amino acids and amines, in the same meteorite extracts. We detected various compound peaks corresponding to amino acids, amines, and their alkylated homologs as unhydrolyzed fractions (Supplementary Fig. 8). This observation is consistent with the amino acid and amine distributions in previous reports (amino acids in Murchison[1]; amino acids in Tagish Lake[2]; amines in Murchison[3]; amines in Tagish Lake[4]). The HPLC/HRMS method applied in this study was not optimized to separate structural isomers of amino acids and amines, however, this analytical procedure enables us to simultaneously confirm the presence of solvent-extractable cyclic and linear N-containing compounds[5].

For comparison with cyclic N-containing compounds mentioned above, Supplementary Figure 9 shows variations in the relative abundances of alkylated amino acid and amine homologs in the meteorite extracts with relevance to the number of carbon atoms in the alkyl groups under the above rough assumptions. The variation of amino acid homologs in Tagish Lake extract is distinct from that in the other two meteorite extracts (Supplementary Fig. 9a), as similarly observed in alkylated imidazole homologs (Fig. 3). In contrast to amino acids and imidazoles, the relative abundances of alkylated amine homologs exponentially decreased with increasing the number of

alkylcarbons (from C_4 to C_7) (Supplementary Fig. 9b). The distinct variation pattern of alkylated amines from that of amino acids and imidazoles might imply their different formation processes during the formation of the early solar system.

Are heavy isotopes suggestive of extraterrestrial origin?

The enrichment of heavy isotopes such as D (^2H), ^{13}C, ^{15}N[refs.6,7] and presence of racemic mixtures of chiral molecules such as amino acids and sugars[8,9] have often been used as evidence of their exogenous origin. Based on the analytical validation of N-heterocyclic compounds using an optimized method (e.g., LC × GC / IRMS)[10], the quantity of nanomoles of nucleobase molecules is promising enough for individual compound-specific stable isotope measurements (e.g., $\delta^{15}N$ of purine and pyrimidine nucleobases) in the context of theoretical values. Based on the concentrations of the detected nucleobases, more than 5 g of meteorite would be necessary for $\delta^{15}N$–isotopic measurements. However, even if nucleobases are extracted from 5 g or more of meteorites, as long as chromatographic separation from other coexisting species at the same retention time is not sufficient, reliable $\delta^{15}N$ values could not be measured, as inferred for the $\delta^{13}C$ of uracil in the Murchison extract[11,12]. A recent laboratory study demonstrated that ^{13}C-enriched organic molecules can be produced with ^{13}C-depleted materials by a kinetic isotope effect during parent-body-like processes, which suggests that heavy C isotope enrichment in extraterrestrial nucleobases may not be indicative of their extraterrestrial

origin[13]. Moreover, because nucleobases are generally not chiral, their origin cannot be identified using this criterion. In contrast, structural diversity, particularly pyrimidine nucleobases, would reasonably be considered an important criterion for suggesting their extraterrestrial origin[14].

Possible scenarios for the synthesis of nucleobases proposed so far

The oligomerization of hydrogen cyanide (HCN) was proposed in the 1960s[15,16] as a possible pathway for the formation of purine nucleobases under prebiotic conditions and has since been substantiated by several experimental studies[16–18]. HCN can be formed by the decomposition of HMT in meteorite parent bodies[5,19] and has been detected in the Murchison meteorite[20] and comets[21], suggesting that the HCN-based pathway could have yielded purine nucleobases in the parent body. Detection in the Murchison #1 extract of 5-amino-4-imidazolecarboxamide (Supplementary Fig. 12), a precursor of guanine, xanthine, and hypoxanthine in the HCN-based purine formation pathways[17], and the expected presence of 5-amino-4-carbonitrile (Supplementary Fig. 13). In addition, the formation of an imidazole structure prior to that of purine is intuitively consistent with the predominance of molecules possessing an imidazole ring (Tables 2 and 3). The detection of 2,4-diaminopyrimidine in Murchison #1 (Supplementary Fig. 3d) and Murray extracts imply the validity of the route for the formation of cytosine and uracil starting from cyanoacetylene (HCCCN)[18]. Although it is unclear whether HCCCN is present in the meteorite parent bodies, it has been detected

in comet 67P[22].

In addition to the HCN-related route, a number of laboratory studies proved that nucleobases can be produced from formamide (NH_2CHO) under the conditions on meteorite parent bodies[23–27]. When NH_2CHO was heated with meteorite powder at around 100 ºC, various kinds of organic molecules including nucleobases were identified in the product[25]. Of note, without meteorite powder, purine was the only product, suggesting a potential role of meteorite powder as catalysts for the synthesis of nucleobases in this experiment[27]. Although the detailed synthesis pathways are not well understood, it is likely that diaminomaleonitrile (DAMN)[26] is a precursor of nucleobase, similar to the case of the HCN-based pathway. Since NH_2CHO has been identified in the interstellar medium[28] and comets[29], it is straightforward that NH_2CHO plays a role as a reactant for the synthesis of nucleobases in meteorites.

Nucleobases can also be synthesized from inorganic materials such as ammonium hydrogen carbonate (NH_4HCO_3) and water through the meteorite impact to the early ocean[30]. After the shock-recovery experiments with the pressures of the samples reaching to 4-7 GPa for 1 μsec, both cytosine and uracil were identified in the product[30].

Detected nitrogen heterocyles

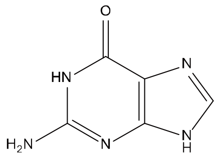

Guanine

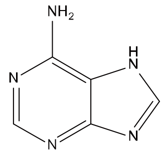

Adenine

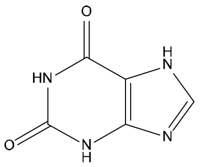

Xanthine

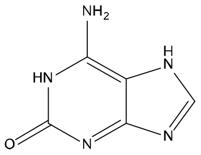

Isoguanine

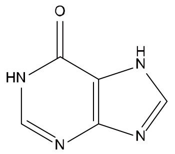

Hypoxanthine

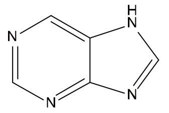

Purine

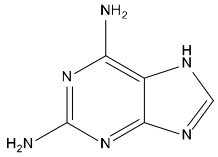

2,6-Diaminopurine

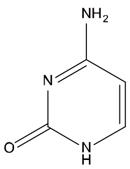

Cytosine

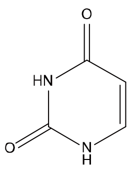

Uracil

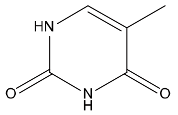

Thymine

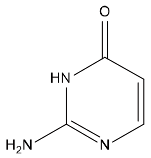

Isocytosine

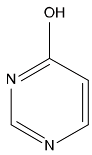

4-Hydroxypyrimidine

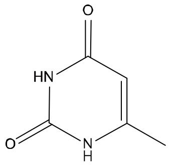

6-Methyluracil

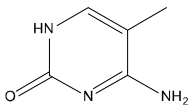

5-Methylcytosine

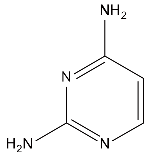

2,4-Diaminopyrimidine

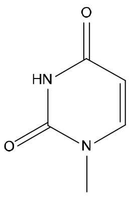

1-Methyluracil

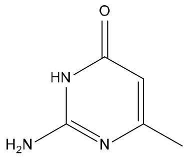

6-Methylisocytosine

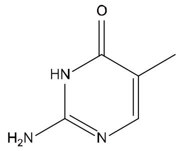

5-Methylisocytosine

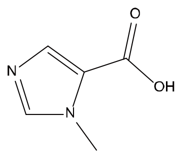

1-Methyl-1H-imidazole
-5-carboxylic acid

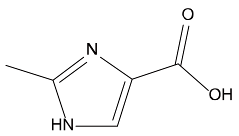

2-Methyl-1H-imidazole
-4-carboxylic acid

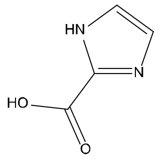

2-Imidazole
carboxylic acid

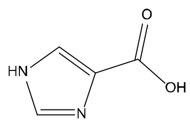

4-Imidazole
carboxylic acid

(Continued)

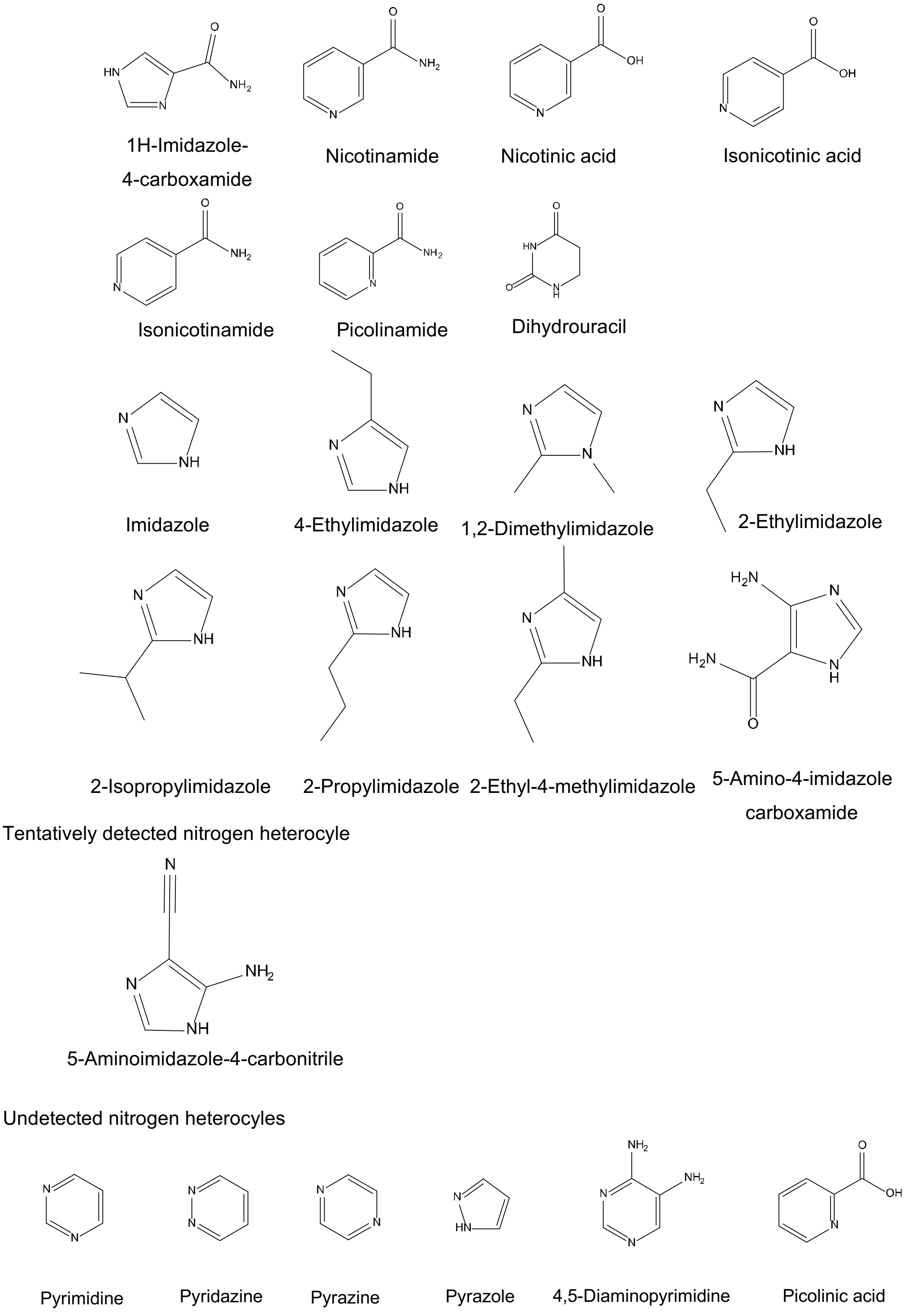

Supplementary Figure 1. Molecular structures of the target molecules in the present study.

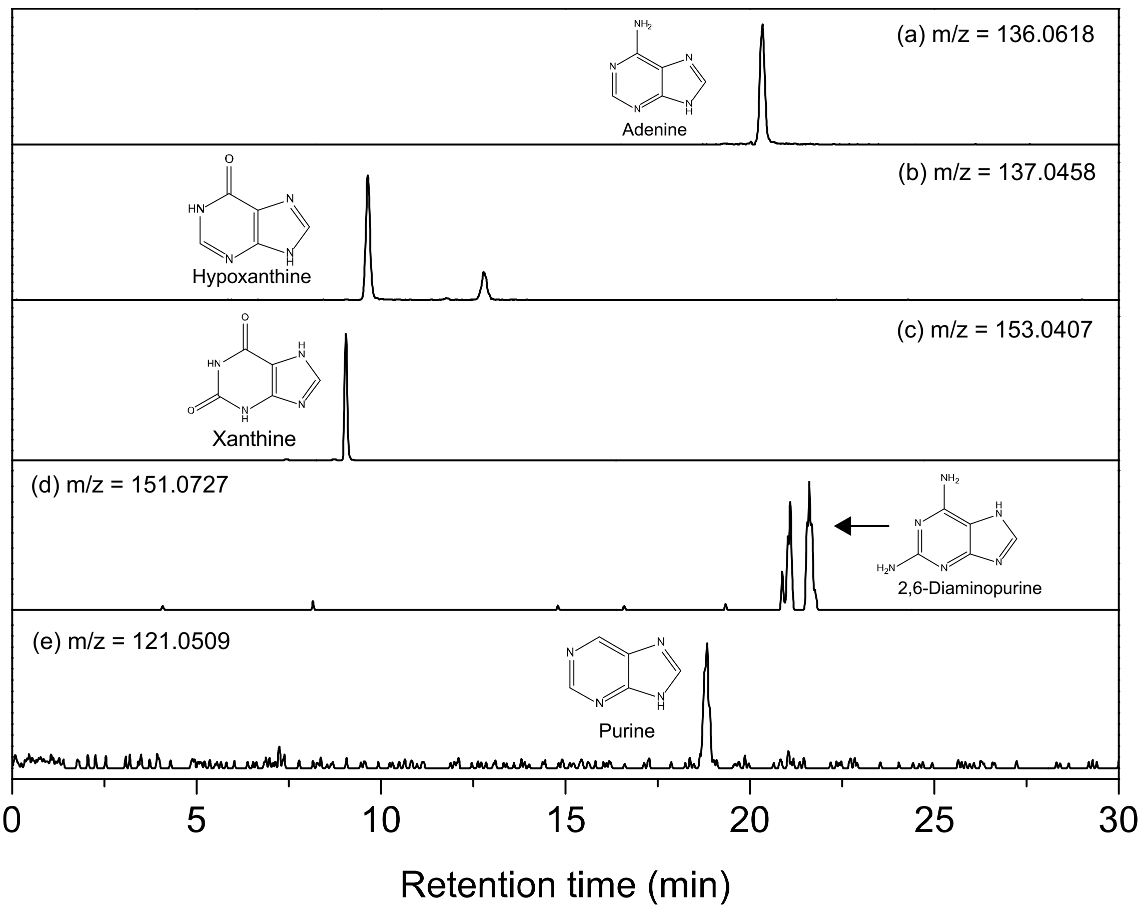

Supplementary Figure 2. Detection of purine nucleobases. Mass chromatograms at **(a)** *m*/*z* = 136.0618 (adenine), **(b)** 137.0458 (hypoxanthine), **(c)** 153.0407 (xanthine), (d) 151.0727 (2,6-diaminopurine, and (e) 121.0509 (purine) within a 3 ppm exact mass window at each monoisotopic mass in the Murchison #1 extract measured using an InertSustain PFP column except purine, which was measured using a Hypercarb column.

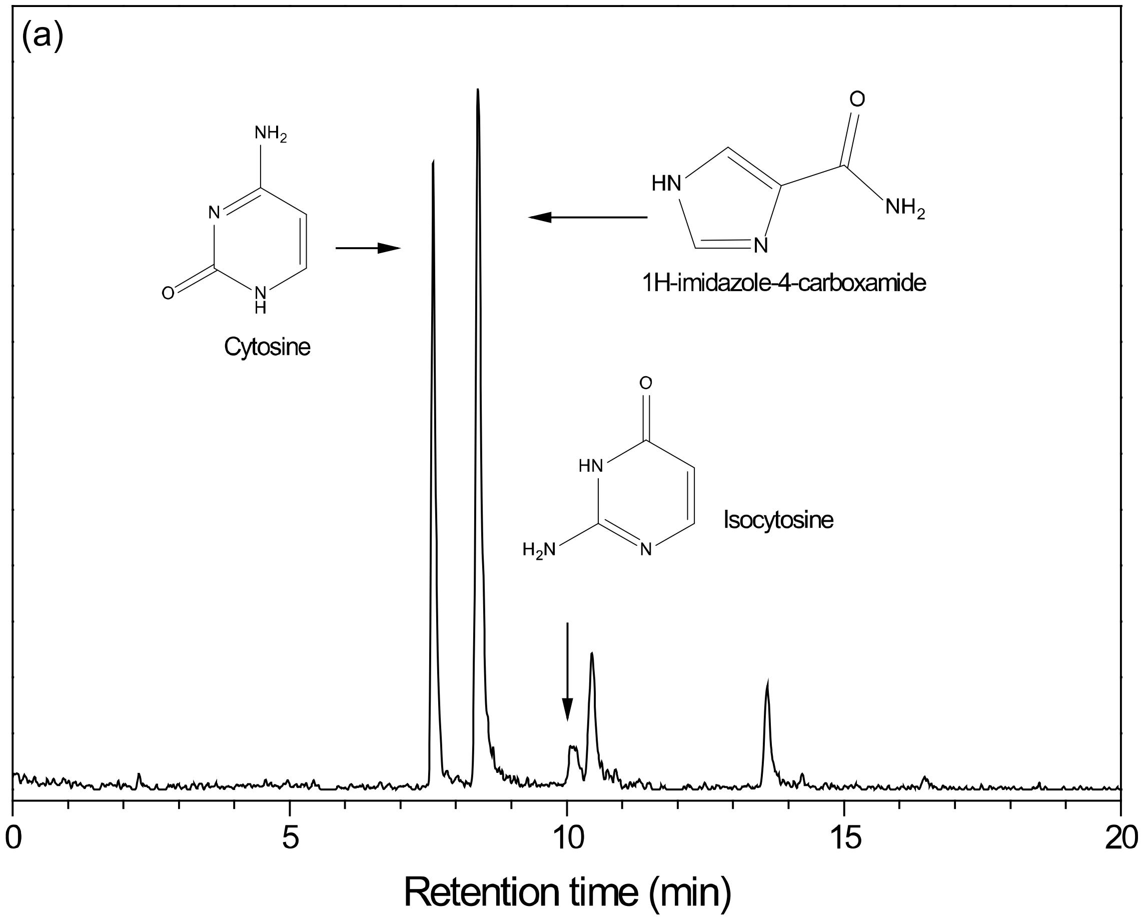

Supplementary Figure 3(a). Detection of cytosine and their isomers. Mass chromatograms at m/z = 112.0505 (cytosine) within a 3 ppm exact mass window at each monoisotopic mass in the Murchison #1 extract measured using a Hypercarb column.

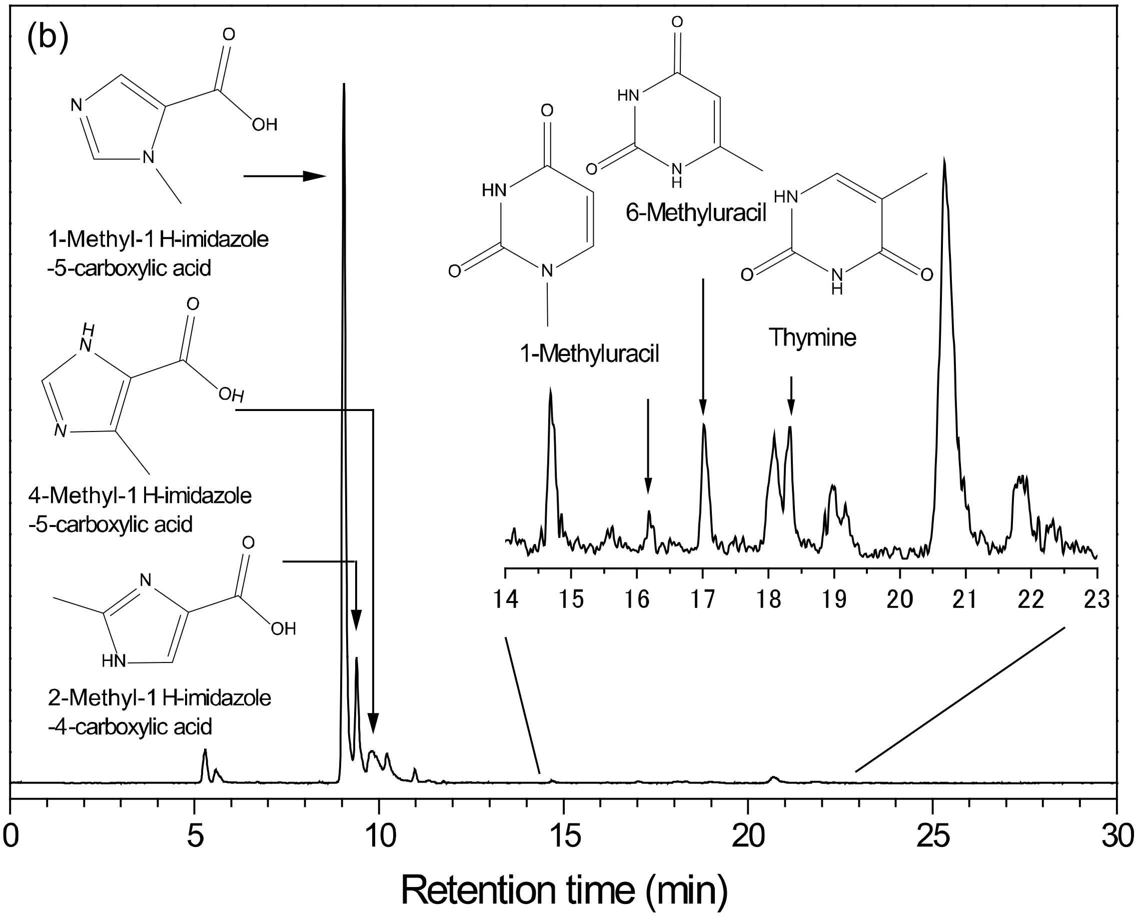

Supplementary Figure 3(b). Detection of thymine and their isomers. Mass chromatograms at *m*/*z* = 127.0502 (thymine) within a 3 ppm exact mass window at each monoisotopic mass in the Murchison #1 extract measured using a Hypercarb column.

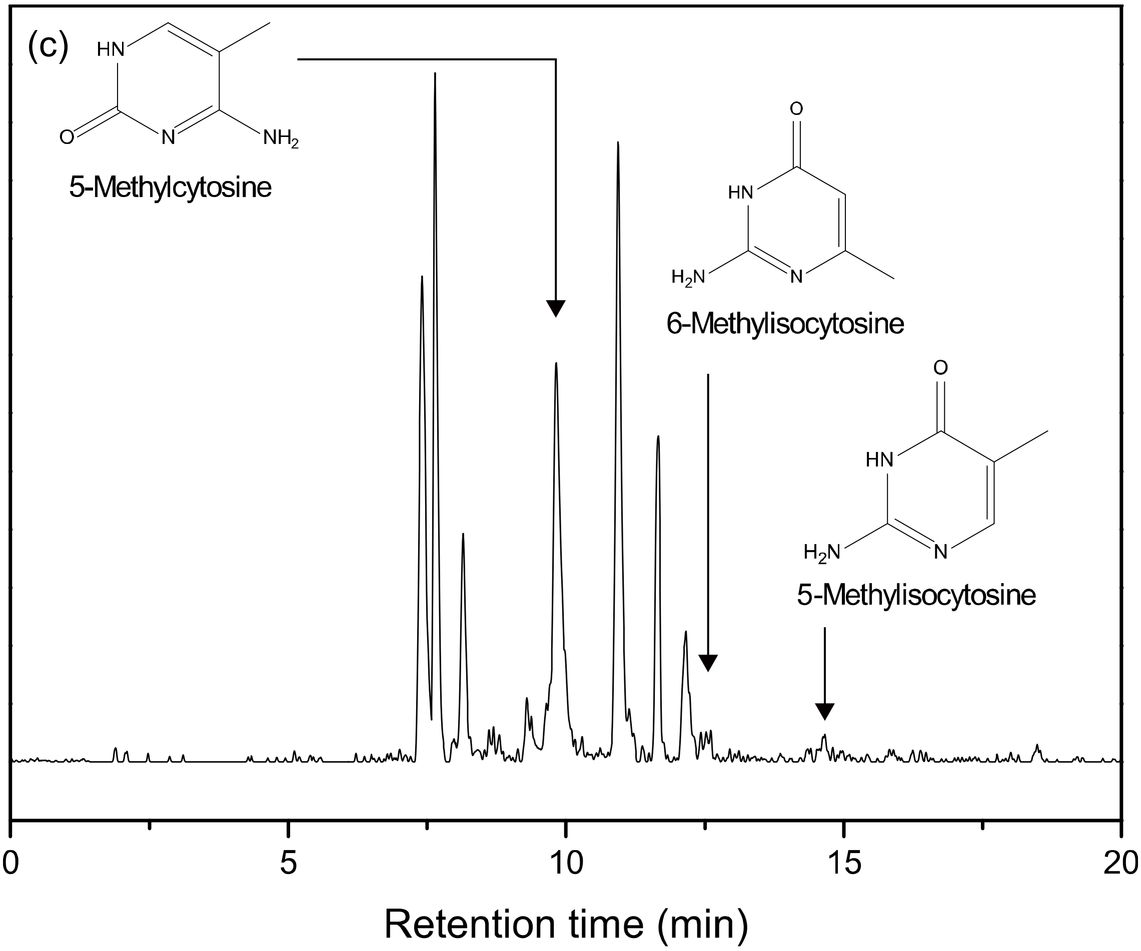

Supplementary Figure 3(c). Detection of 5-methylcytosine and their isomers. Mass chromatograms at *m*/*z* = 126.0662 (5-methylcytosine) within a 3 ppm exact mass window at each monoisotopic mass in the Murchison #1 extract measured using a Hypercarb column.

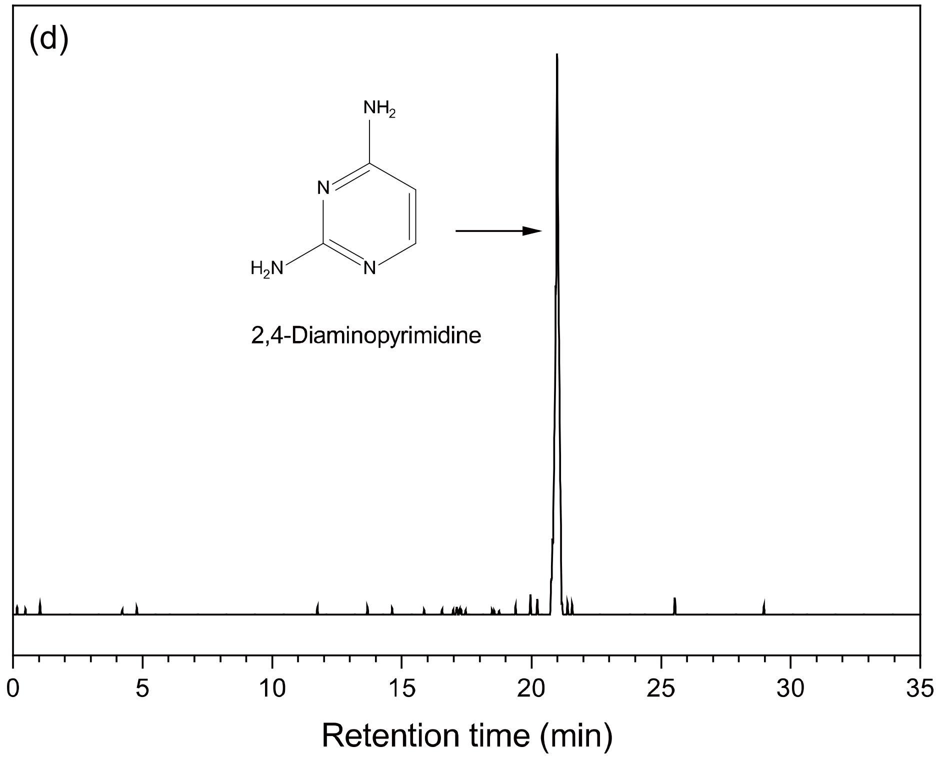

Supplementary Figure 3(d). Detection of 2,4-diaminopyrimidine. Mass chromatograms at *m*/*z* = 111.0665 (2,4-diaminopyrimidine) within a 3 ppm exact mass window at each monoisotopic mass in the Murchison #1 extract measured using an InertSustain PFP column.

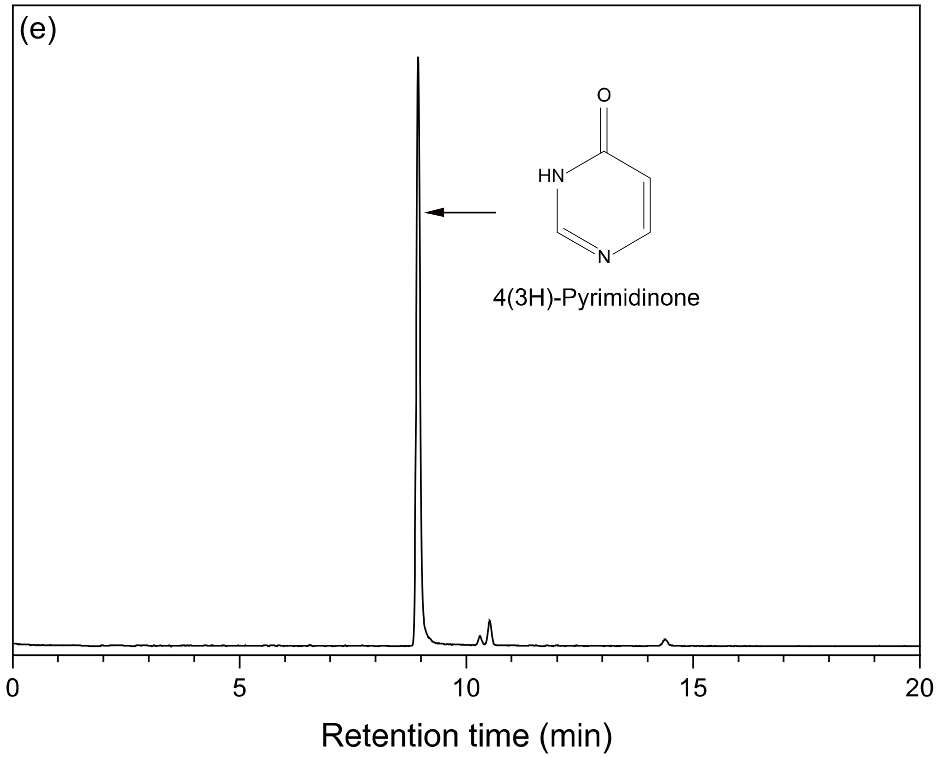

Supplementary Figure 3(e). Detection of 4(3H)-pyrimidinone (4-hydroxypyrimidine). Mass chromatograms at *m*/*z* = 97.0396 (4-hydroxypyrimidine) within a 3 ppm exact mass window at each monoisotopic mass in the Murchison #1 extract measured using a Hypercarb column.

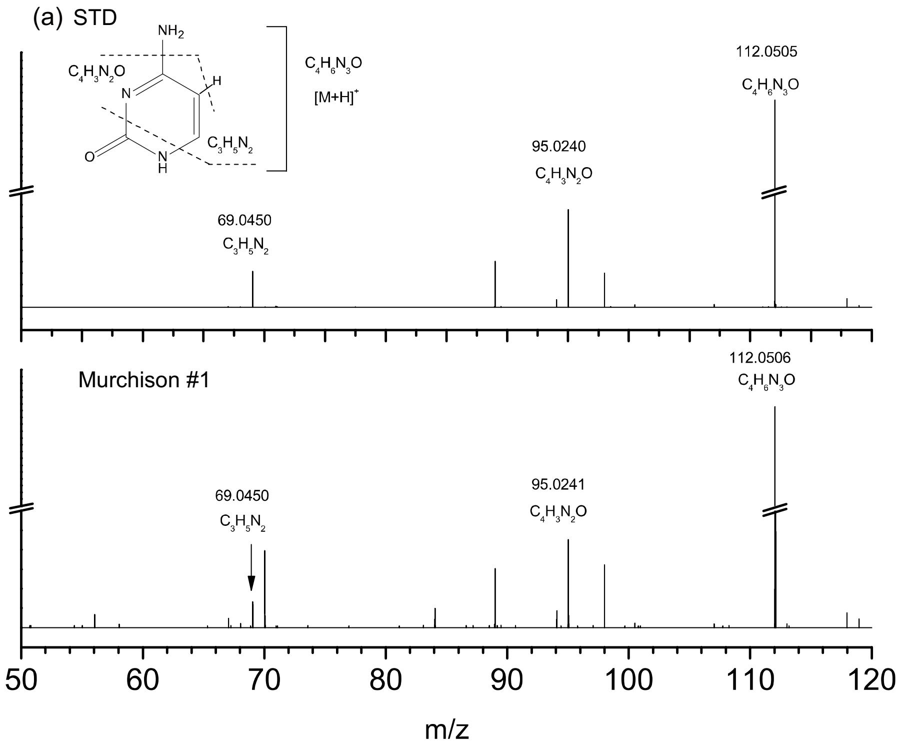

Supplementary Figure 4(a). MS/MS measurements of cytosine. Mass fragmentation patterns of cytosine STD and cytosine detected in the Murchison #1 extract measured by MS/MS experiments.

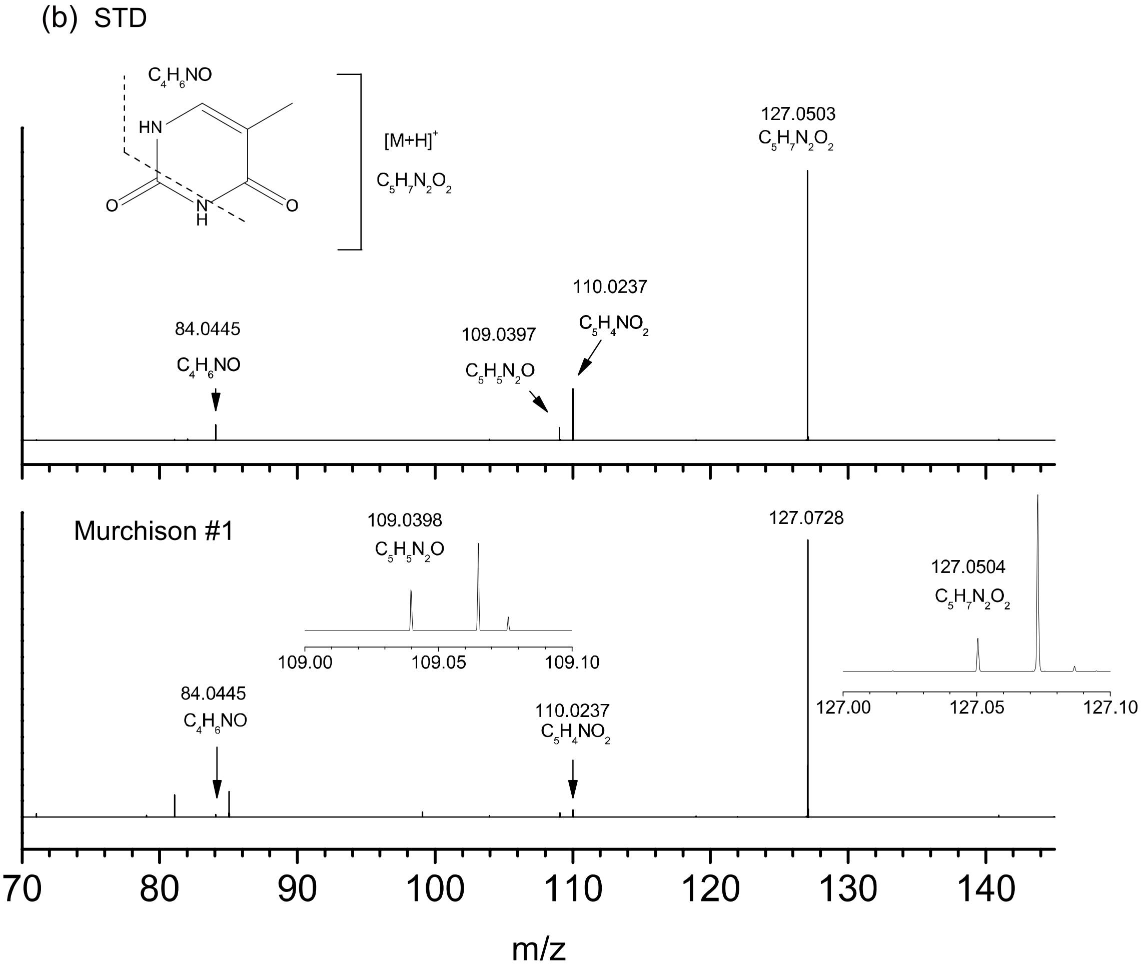

Supplementary Figure 4(b). MS/MS measurements of thymine. Mass fragmentation patterns of thymine STD and cytosine detected in the Murchison #1 extract measured by MS/MS experiments.

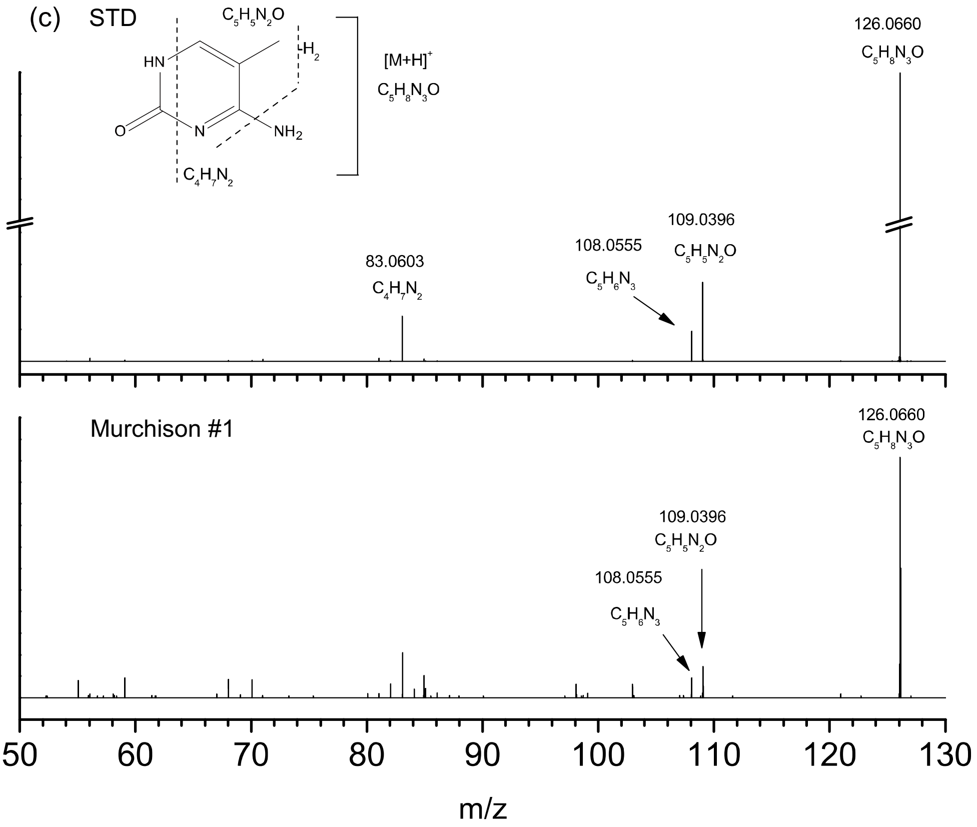

Supplementary Figure 4(c). MS/MS measurements of 5-methylcytosine. Mass fragmentation patterns of 5-methylcytosine STD and cytosine detected in the Murchison #1 extract measured by MS/MS experiments.

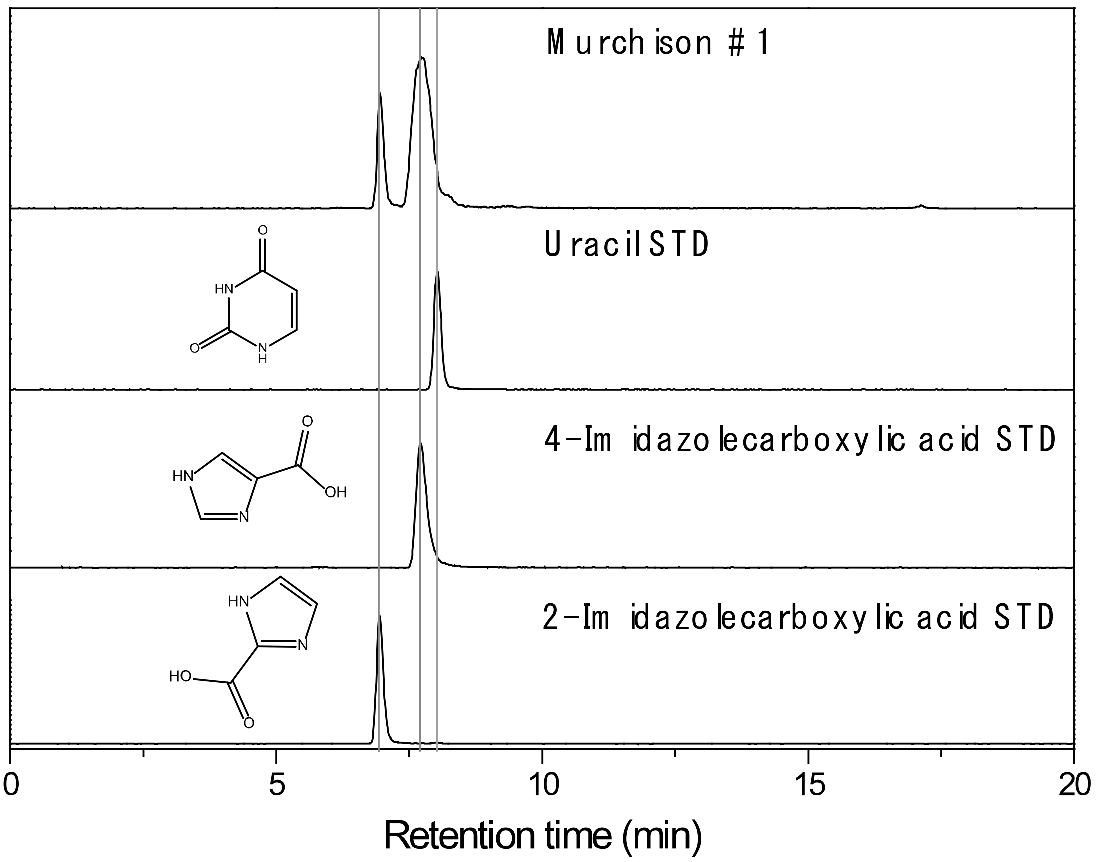

Supplementary Figure 5. Insufficient peak separation for uracil isomers. (From top to the bottom) Mass chromatograms at m/z = 113.0346 in the Murchison #1 extract, uracil standard reagent (STD), and 4-imidazolecarboxylic acid STD measured using an InertSustain PFP column. Since the uracil and 4-imidazolecarboxylic acid peaks overlap on the Murchison #1 mass chromatogram, their precise identification and quantification was not possible.

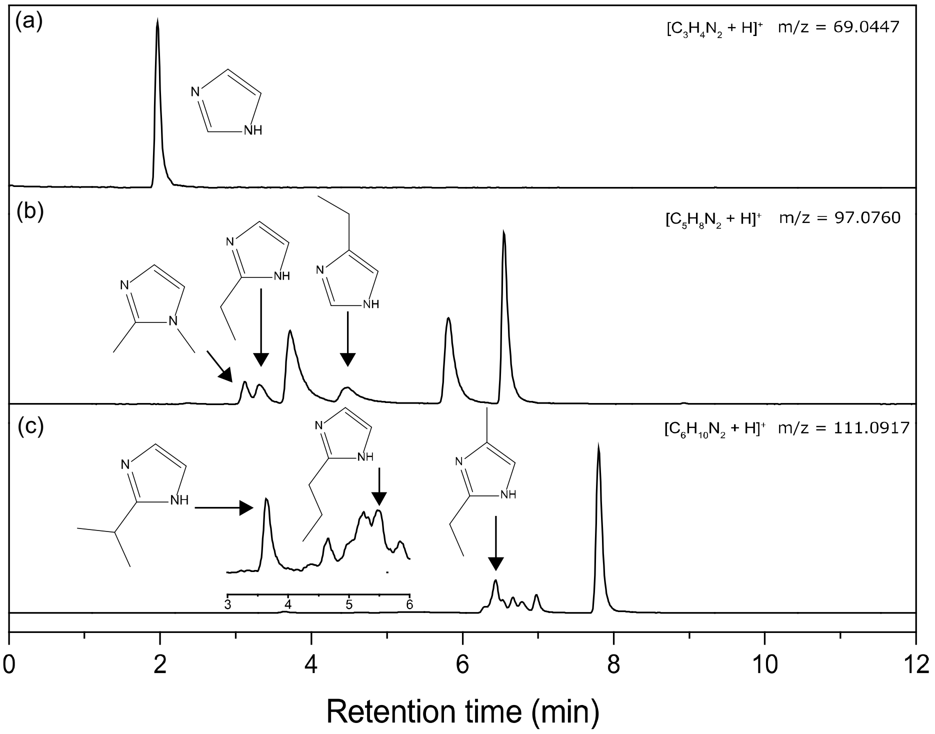

Supplementary Figure 6. Identification of imidazole and its alkylated analogues. Mass chromatograms at *m/z* = **(a)** 69.0447 (imidazole), **(b)** 97.0760 (C_2-alkylimidazoles), and **(c)** 111.0917 (C_3-alkylimidazoles) in the Murchison #1 extract using a Hypercarb column, where the subscript *n* represents the number of carbon atoms in the alkyl carbon chain(s) attached to the imidazole ring. C_1-alkylimidazoles could not be measured since their *m/z* (83.0604, identical to the *m/z* of the acetonitrile dimer) is used as a Lockmass in the HPLC/HRMS measurement.

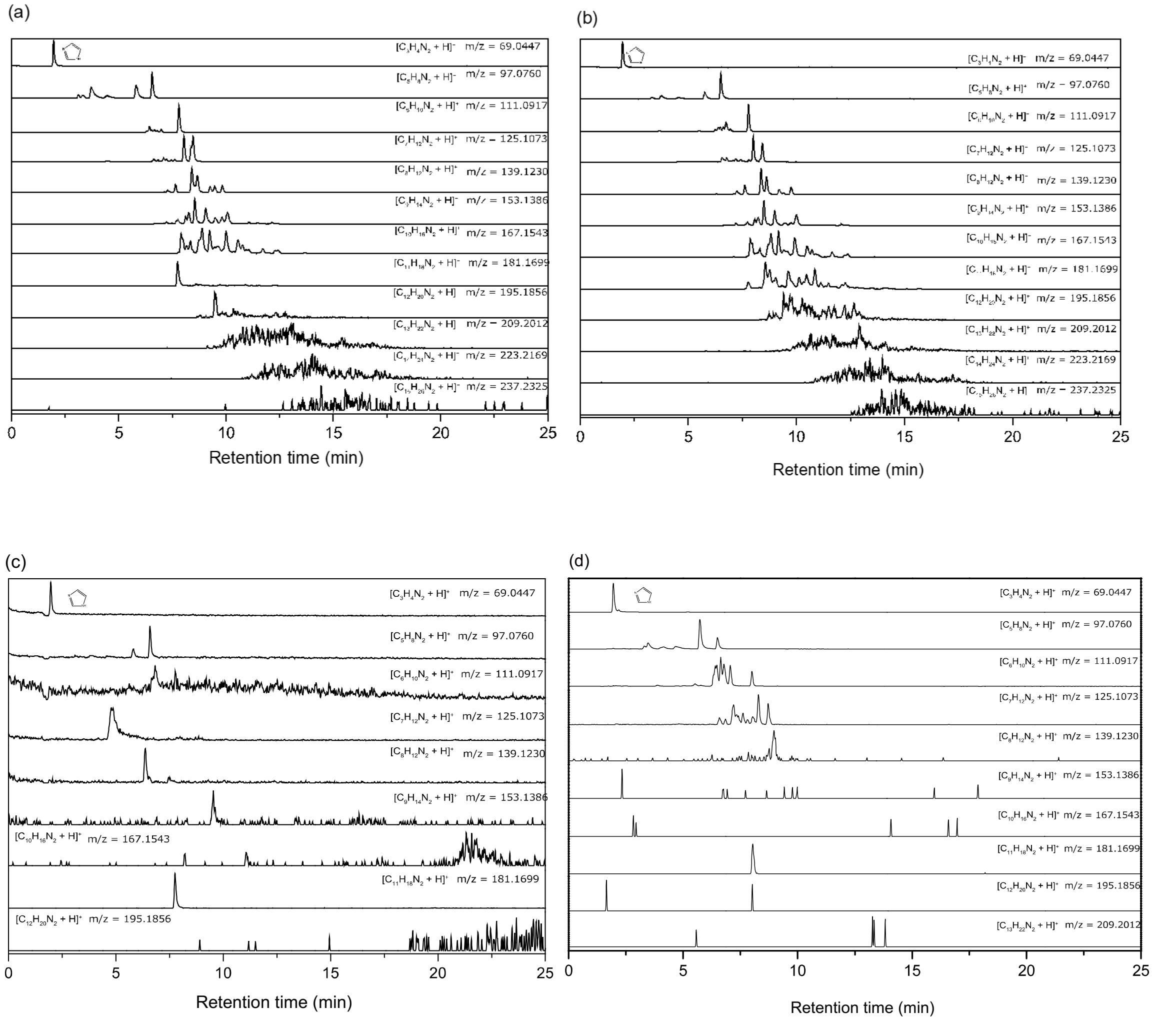

Supplementary Figure 7. Alkylimidazole analogues in meteorites. Mass chromatograms at *m*/*z* corresponding to imidazole and its alkylated homologues, with the number of alkylcarbons up to 12, in the **(a)** Murchison #1, **(b)** Murray, **(c)** Tagish Lake meteorites, and **(d)** the organic residues formed by photochemical reactions of interstellar ice analogues containing water, carbon monoxide, methanol, and ammonia, using a Hypercarb column. The dataset **(d)** was obtained by reanalysis of our previous data reported in Oba et al.[5]

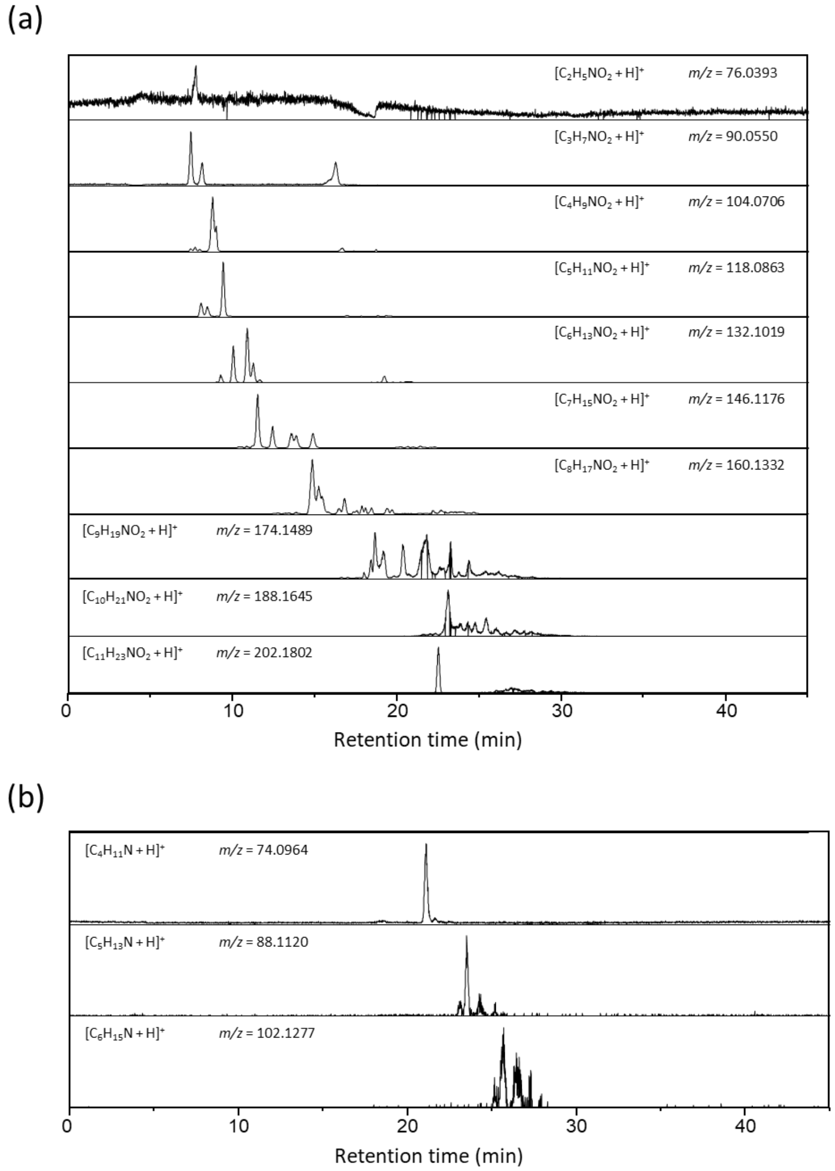

Supplementary Figure 8. Possible detection of alkylated molecules. **(a)** Representative mass chromatograms at *m*/*z* corresponding to glycine (top) and its alkylated homologues (within a 3 ppm exact mass window at the monoisotopic mass), with the number of alkylcarbons up to 11, in the Murchison #1 meteorite measured using an InertSustain PFP column. **(b)** Representative mass chromatograms at *m*/*z* corresponding to butylamine (top) and its alkylated homologues (within a 3 ppm exact mass window at the monoisotopic mass), with the number of alkylcarbons up to 6, in the Murchison #1 meteorite measured using an InertSustain PFP column. Methylamine and Ethylamine could not be analyzed by the HPLC/HRMS method applied in this study because of mass cut-off for masses below *m/z* 50.

(a)

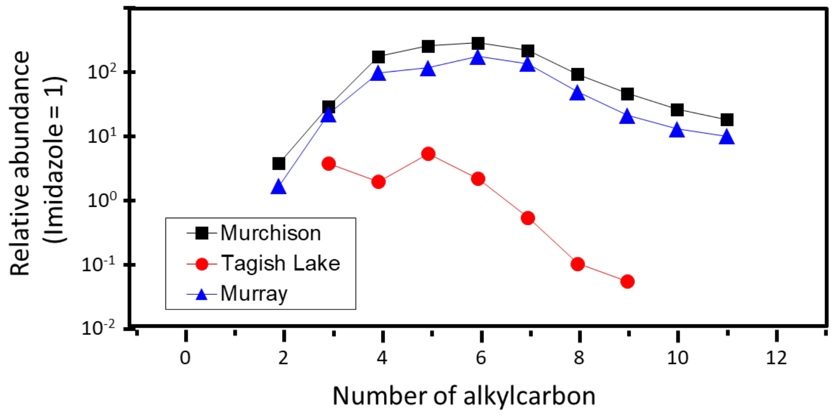

(b)

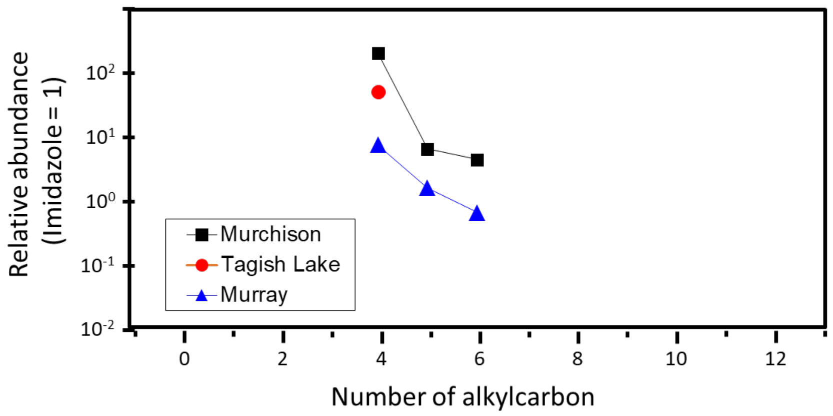

Supplementary Figure 9. Variations in the hypothesized relative abundances of molecules. **(a)** amino acids or **(b)** amines and their alkylated homologues in the Murchison #1, Tagish Lake, and Murray extracts, with relevance to the number of alkylcarbons. The relative abundances were estimated by dividing the peak area of each species by that of imidazole in the same meteorite (Supplementary Figure 7).

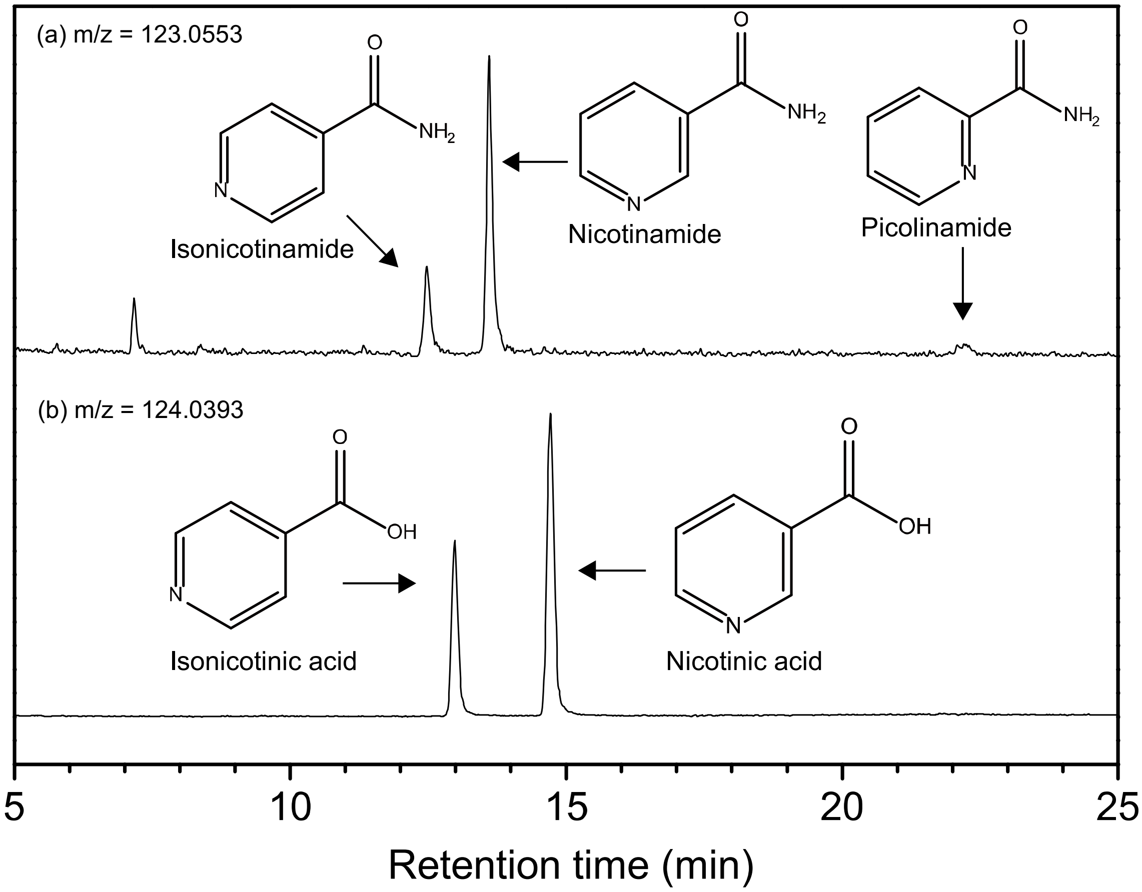

Supplementary Figure 10. Detection of nicotinic acid, nicotinamide and their isomers. Mass chromatograms at **(a)** *m/z* = 123.0553 and **(b)** *m/z* = 124.0393 for the protonated ions of Mass chromatograms at *m*/*z* = 123.0553 (upper) and 124.0393 (lower) corresponding to the protonated ions of nicotinamide and nicotinic acid, respectively, and their structural isomers within a 3 ppm exact mass window at each monoisotopic mass in the Murchison #1 extract. These molecules were measured using a Hypercarb column. Since picolinic acid was not detected under the present analytical conditions, no peak in the chromatogram does not necessarily mean its absence in meteorites.

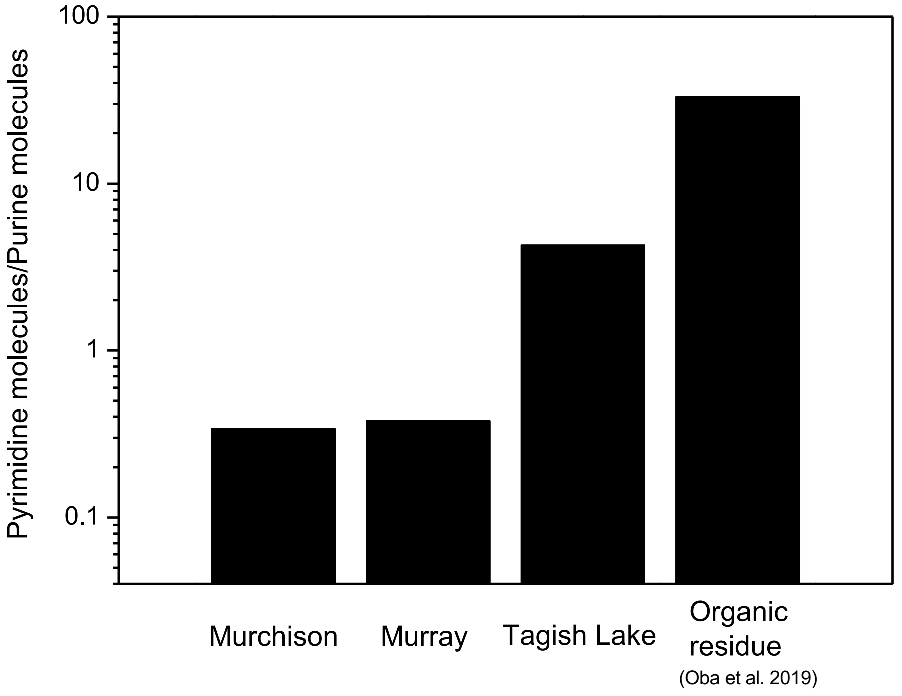

Supplementary Figure 11. Variations in pyrimidine/purine molecules ratio. Relative abundances of pyrimidine molecules and purine molecules detected in the meteorite extracts and in the organic residues produced by photolysis of interstellar ice analogs at 10K[5].

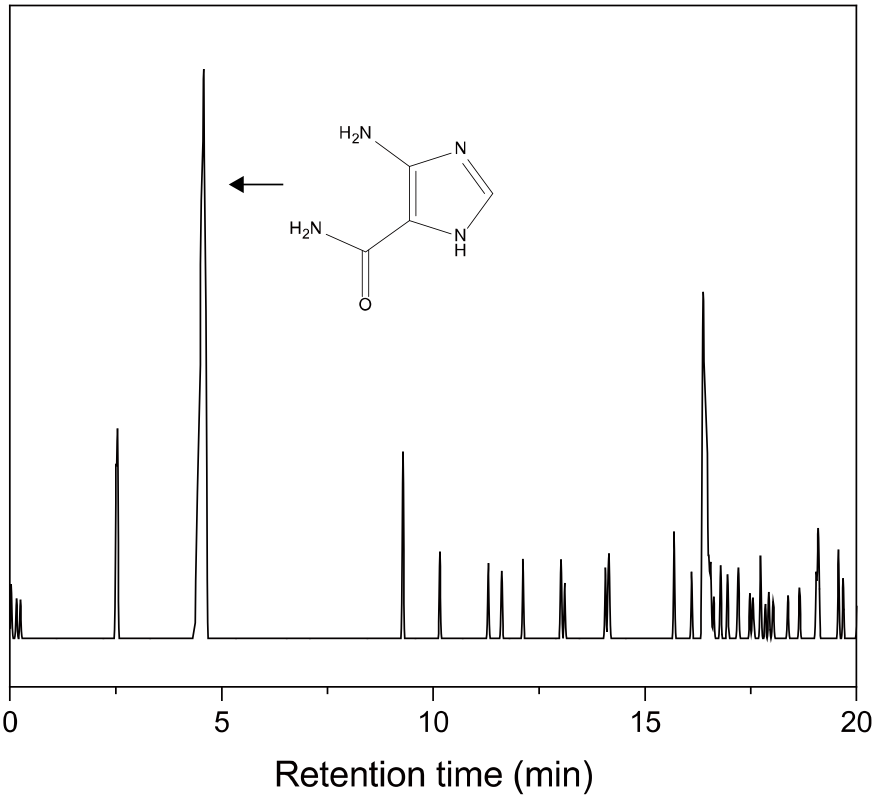

Supplementary Figure 12. Detection of 5-amino-4-imidazolecarboxamide. Mass chromatogram at *m*/*z* = 127.0614 for the negative ion of 5-amino-4-imidazolecarboxamide in the Murchison #1 extract measured using a HILICpack VG-50 column.

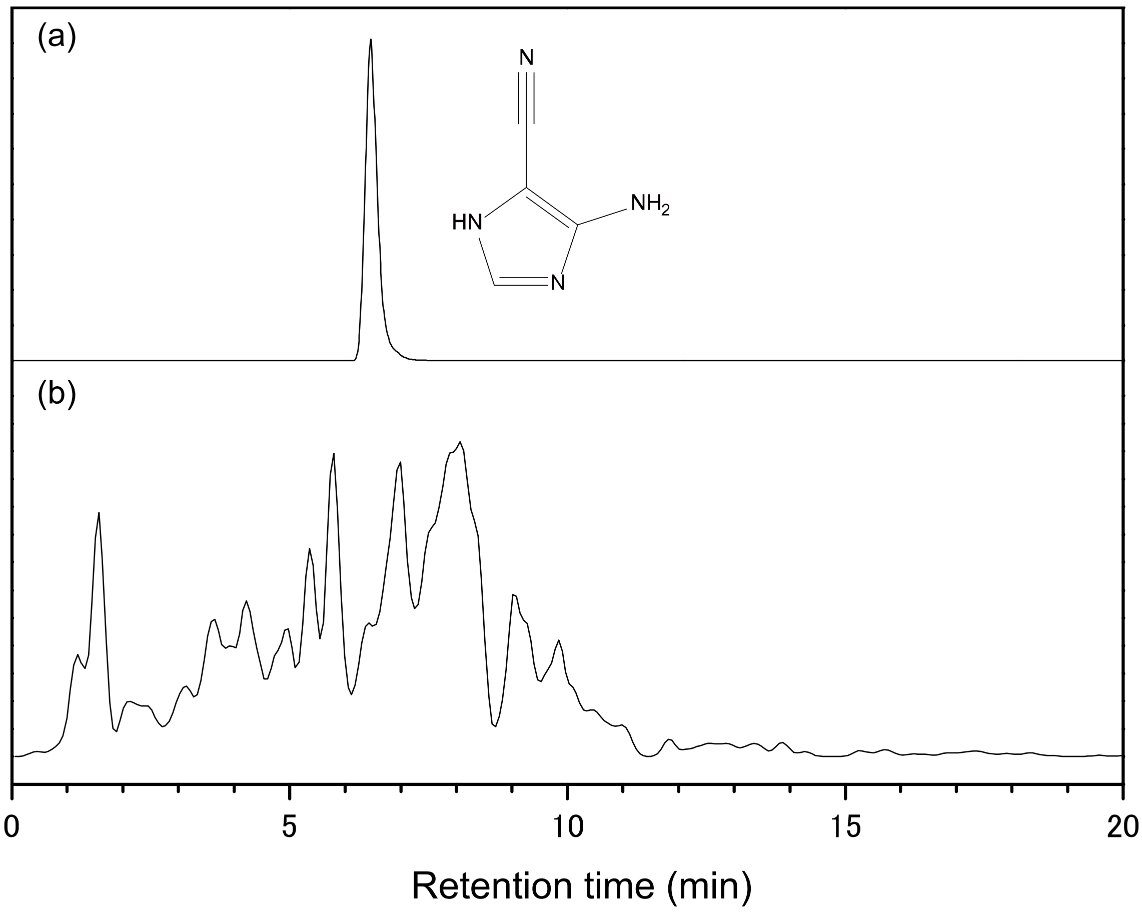

Supplementary Figure 13. Possible presence of 5-aminoimidazole-4-carbonitile in meteorites. Mass chromatograms at *m/z* = 107.0363 within a 3 ppm exact mass window at each monoisotopic mass for **(a)** the 5-aminoimidazole-4-carbonitrile standard reagent and **(b)** the Murchison #1 extract measured using a HILICpack VG-50 column in negative ion mode. Peak separation was inadequate to identify 5-aminoimidazole-4-carbonitrile in the Murchison #1 extract, but we strongly expect its presence in the extract.

Supplementary Table 1. Representative values of the detected fragment (*m/z*) after MS/MS experiments for pyrimidine nucleobases and their structural isomers.

Molecular name	Molecular formula	*m/z* $[M+H]^+$	Detected fragment (*m/z*)
Cytosine	$C_4H_5N_3O$	112.0505	112.0505 95.0240 69.0450
Isocytosine	$C_4H_5N_3O$	112.0505	112.0506 98.0073 95.0241 70.0290
1H-Imidazole-4-carboxamide	$C_4H_5N_3O$	112.0505	112.0507 95.0241
Uracil	$C_4H_4N_2O_2$	113.0346	113.0346 96.0080 79.0290
Imidazole-4-carboxylic acid	$C_4H_4N_2O_2$	113.0346	113.0346 99.0061 95.0241 71.0945 70.0653 68.0498
Imidazole-2-carboxylic acid	$C_4H_4N_2O_2$	113.0346	113.0345
5-Methylcytosine	$C_5H_7N_3O$	126.0662	126.0660 109.0396 108.0555 83.0603
6-Methylisocytosine	$C_5H_7N_3O$	126.0662	126.0661 84.0444
Thymine	$C_5H_6N_2O_2$	127.0502	127.0503 110.0237 109.0397 84.0445
1-Methyluracil	$C_5H_6N_2O_2$	127.0502	127.0501 84.0444 70.0289
6-Methyluracil	$C_5H_6N_2O_2$	127.0502	127.0503 110.0238 84.0445
1-Methyl-1H-imidazole-5-carboxylic acid	$C_5H_6N_2O_2$	127.0502	127.0501 83.0604
2-Methyl-1H-imidazole-4-carboxylic acid	$C_5H_6N_2O_2$	127.0502	127.0500
4-Methyl-1H-imidazole-5-carboxylic acid	$C_5H_6N_2O_2$	127.0502	127.0502

Supplementary Table 2. Acid to amide ratio. Relative abundance of nicotinic acid to nicotinamide in the meteorite extracts and photochemical product at 10K.

Sample	Acid/amide ratio	
	Nicotinic acid/Nicotinamide	Isonicotinic acid/Isonicotinamide
Murchison #1	9.3	20.5
Murchison #2	13.1	72.8
Tagish Lake	20.1	33.2
Murray	9.6	48.3
Photochemical products*	0.8	1.0

*Please see Oba et al.[5]

Supplementary Table 3. Pyrimidines and purines in organic residues. Relative abundances of pyrimidine and purine molecules in organic residues produced by photochemical reactions of interstellar ice analogs (Reanalysis of the sample produced in Oba et al.[5]).

Detected molecules	Relative abundance (cytosine = 1)	Pyrimidine/purine ratio
Pyrimidine molecules		
Cytosine	1	
Thymine	7.0×10^{-1}	
Uracil	6.5×10^{-1}	
4-Hydroxypyrimidine	4.3	
5-Methylcytosine	8.9	
Purine molecules		
Adenine	5.0×10^{-2}	
Hypoxanthine	3.0×10^{-2}	
Xanthine	2.0×10^{-2}	
Purine	3.7×10^{-1}	
		33.1

Supplementary References

1. Glavin, D. P. et al. Amino acid analyses of Antarctic CM2 meteorites using liquid chromatography-time of flight-mass spectrometry. *Meteor. Planet. Sci.* **41**, 889–902 (2006).
2. Glavin, D. P. et al. Unusual nonterrestrial l-proteinogenic amino acid excesses in the Tagish Lake meteorite. *Meteor. Planet. Sci* **47**, 1347–1364 (2012).
3. Aponte, J. C., Dworkin, J. P. & Elsila, J. E. Assessing the origins of aliphatic amines in the Murchison meteorite from their compound-specific carbon isotopic ratios and enantiomeric composition. *Geochim. Cosmochim. Acta* **141**, 331–345 (2014).
4. Pizzarello, S. et al., The Organic Content of the Tagish Lake Meteorite. *Science* **293**, 2236–2239 (2001).
5. Oba, Y., Takano, Y., Naraoka, H., Watanabe, N. & Kouchi, A. Nucleobase synthesis in interstellar ices. *Nature Commun.* **10**, 4413 (2019).
6. Kerridge, J. F., Chang, S. & Shipp, R. Isotopic characterisation of kerogen-like material in the Murchison carbonaceous chondrite. *Geochim. Cosmochim. Acta* **51**, 2527–2540 (1987).
7. Sephton, M. A. & Botta, O. Extraterrestrial organic matter and the detection of life. *Space Sci. Rev.* **135**, 25–35 (2008).

8. Furukawa Y. et al., Extraterrestrial ribose and other sugars in primitive meteorites. *Proc. Natl. Acad. Sci. U.S.A.* **116**, 24440–24445 (2019).

9. Cronin, J. R. & Pizzarello, S. Enantiomeric excesses in meteoritic amino acids. *Science* **275**, 951–955 (1997).

10. Takano, Y. Chikaraishi, Y. & Ohkouchi, N. Isolation of underivatized amino acids by ion-pair high performance liquid chromatography for precise measurement of nitrogen isotopic composition of amino acids: Development of comprehensive LC × GC/C/IRMS method. *Int. J. Mass. Spectrom.* **379**, 16–25 (2015).

11. Martins, Z. et al., Extraterrestrial nucleobases in the Murchison meteorite. *Earth. Planet. Sci. Lett.* **270**, 130–136 (2008).

12. Callahan, M. P. et al., Carbonaceous meteorites contain a wide range of extraterrestrial nucleobases. *Proc. Natl. Acad. Sci. U.S.A.* **108**, 13995–13998 (2011).

13. Furukawa, Y., Iwasa, Y. & Chikaraishi, Y. Synthesis of ^{13}C-enriched amino acids with ^{13}C-depleted insoluble organic matter in a formose-type reaction in the early solar system. *Sci. Adv.* **7,** eabd3575 (2021).

14. Schmitt-Kopplin, P. et al., High molecular diversity of extraterrestrial organic matter in Murchison meteorite revealed 40 years after its fall. *Proc. Natl. Acad. Sci. U.S.A.* **107**, 2763–2768 (2010).

15. Oró, J. Mechanism of synthesis of adenine from hydrogen cyanide under possible primitive Earth conditions. *Nature* **191**, 1193–1194 (1961).

16. Sanchez, R. A., Ferris, J. P. & Orgel, L. E. Studies in prebiotic synthesis. II. Synthesis of purine precursors and amino acids from aqueous hydrogen cyanide. *J. Mol. Biol.* **30**, 223–253 (1967).

17. Cleaves II, H. J. "Nucleobases on the primitive Earth: Their sources and stabilities" in Prebiotic Chemistry and Chemical Evolution of Nucleic Acids, Menor-Salván, C. ed. (Springer, 2018), pp. 1–19.

18. Menor-Salván, C. "From the dawn of organic chemistry to astrobiology: Urea as a foundational component in the origin of nucleobases and nucleotides" in Prebiotic Chemistry and Chemical Evolution of Nucleic Acids, Menor-Salván, C. ed. (Springer, 2018), pp. 85–142.

19. Bernstein, M. P., Sandford, S. A., Allamandola, L. J., Chang, S. & Scharberg, M. A. Organic compounds produced by photolysis of realistic interstellar and cometary ice analogs containing methanol. *Astrophys. J.* **454**, 327–344 (1995).

20. Pizzarello, S. Hydrogen cyanide in the Murchison meteorite. *Astrophys. J. Lett.* **754**, L27 (2012).

21. Bockelée-Morvan, D. & Biver, N. The composition of cometary ices. *Phil. Trans. R.*

Soc. A **375**, 20160252 (2017).

22. Rubin, M. et al., Elemental and molecular abundances in comet 67P/Churyumov-Gerasimenko. *Mon. Not. R. Astron. Soc.* **489**, 594–607 (2019).

23. Saladino, R. et al., Meteorite-catalyzed syntheses of nucleosides and of other prebiotic compounds from formamide under proton irradiation. *Proc. Natl. Acad. Sci. U.S.A.* **112**, E2746–E2755 (2015).

24. Ferus, M. et al., High-energy chemistry of formamide: A simpler way for nucleobase formation. *J. Phys. Chem.* A **118,** 719–736 (2014).

25. Saladino, R., Botta, G., Delfino, M. & Di Mauro, E. Meteorites as catalysts for prebiotic chemistry. *Chem. A Eur. J.* **19**, 16916–16922 (2013).

26. Ferus, M. et al., High-energy chemistry of formamide: A unified mechanism of nucleobase formation. *Proc. Natl. Acad. Sci. U. S. A.* **112**, 657–663 (2015).

27. Saladino, R. et al., One-pot TiO_2-catalyzed synthesis of nucleic bases and acyclonucleosides from formamide: Implications for the origin of life. *ChemBioChem* **4**, 514–521 (2003).

28. López-Sepulcre, A. et al., Interstellar formamide (NH_2CHO), a key prebiotic precursor. *ACS Earth Space Chem.* **3**, 2122–2137 (2019).

29. Bockelée-Morvan, D. et al., New molecules found in comet C/1995 O1 (Hale-Bopp)

Investigating the link between cometary and interstellar material. *Astron. Astrophys.* **353,** 1101–1114 (2000).

30. Furukawa, Y., Nakazawa, H., Sekine, T., Kobayashi, T. & Kakegawa, T. Nucleobase and amino acid formation through impacts of meteorites on the early ocean. *Earth Planet. Sci. Lett.* **429**, 216–222 (2015).
